# Naturalistic investigation of cannabis strains varying in THC and CBD ratios and verbal recognition memory

**DOI:** 10.3389/fpsyg.2025.1685412

**Published:** 2026-01-06

**Authors:** Katie N. Paulich, Christian Place, Gregory Giordano, William B. Carpenter, Tim Curran, L. Cinnamon Bidwell

**Affiliations:** 1 Institute of Cognitive Science, University of Colorado Boulder, Boulder, CO, United States; 2 Department of Psychology and Neuroscience, University of Colorado Boulder, Boulder, CO, United States

**Keywords:** cannabis, THC, CBD, recognition memory, mobile laboratory, naturalistic design

## Abstract

**Introduction:**

Cannabis, which contains cannabinoids delta-9-tetrahydrocannabinol (THC) and cannabidiol (CBD), has become the third-most-commonly used psychoactive substance in the United States. The legal market has grown alongside the increase in adult cannabis use, resulting in greater availability of products with a wide range of THC and CBD content. One of the most consistently observed cognitive effects of cannabis use is impairment of verbal memory. Although previous research shows that THC reduces accuracy in verbal memory tasks, less is known about potential differential acute effects of THC and CBD on recognition memory. Previous studies have suggested a protective effect of CBD (when combined with THC) on recognition memory, though research is mixed.

**Methods:**

In the current study, we hypothesized that THC would result in lower recognition memory accuracy, and that when paired with CBD, the CBD would reduce the effects of THC on memory. Participants were randomly assigned to one of three legal market strains of flower product with varying THC:CBD ratios: a THC-dominant strain (*N* = 40); a roughly 1:1 THC:CBD strain (*N* = 38); and a CBD-dominant strain (*N* = 38). Participants completed two experimental sessions in which they either (a) used their assigned strain ad libitum in their private residence, or (b) did not use cannabis prior to completing a recognition memory task in our mobile laboratory.

**Results:**

We found that participants assigned to the THC-based strain in the cannabis use condition demonstrated lower recognition memory accuracy, more false alarms, a more liberal response bias, and slower reaction time. In contrast, participants assigned to the 1:1 THC:CBD strain had no significant recognition memory impairment, supporting our hypothesis that CBD may reduce the effects of THC on memory.

**Discussion:**

Given that adult cannabis use has become more prevalent, our results have implications to public health. For individuals who use primarily THC-based products, strains that also contain CBD may hold harm reduction potential, as CBD could help mitigate the impact of THC on recognition memory. Notably, bodily sensations of intoxication did not differ significantly between THC-dominant strains with varying amounts of CBD.

## Introduction

As of 2025, cannabis is legal in 39 states for medical use and 24 states for recreational use. Adult cannabis use has increased in recent years alongside the growing legal market, and cannabis has become the third-most-commonly used psychoactive substance in the United States after alcohol and cigarettes (Carliner et al., 2017). Cannabis contains many cannabinoids, the most widely recognized and studied of which are delta-9-tetrahydrocannabinol (THC), the primary psychoactive plant compound, and cannabidiol (CBD).

Previous studies have found that cannabis use impairs memory while under the influence (Bossong et al., 2014; Broyd et al., 2016; Schoeler and Bhattacharyya, 2013). Specifically, the acute use of low-percentage THC has been found to reduce accuracy in verbal recognition tasks in addition to reducing both accuracy and reaction time in working memory tasks (Ilhan et al., 2004). A review of the literature on acute cannabis use also found that THC transiently impaired immediate and free recall in a variety of studies (Ranganathan and D’Souza, 2006). Yet, less is known about potential differential effects of THC and CBD on memory. A previous study found that CBD reduced THC-related episodic memory impairments (Englund et al., 2013). However, a recent study by the same author found that although THC was associated with delayed verbal recall, CBD did not modulate the effect of THC on verbal recall (Englund et al., 2023). Furthermore, Morgan et al. (2018) found that THC impaired episodic and working memory, but the co-administration of CBD did not attenuate this effect. Another study found that high-potency cannabis flower with CBD impaired free recall, and high-potency flower without CBD impaired source memory (Cuttler et al., 2021). However, to our knowledge, few studies exist on the differential effects of THC and CBD on recognition memory specifically. Preliminary data from our lab suggest that THC impaired verbal recognition memory, while the combination of CBD and THC was not associated with impairment (Curran T. et al., 2020), indicating a potentially protective effect of CBD. This potentially protective effect of CBD on recognition memory may have broad implications and is a topic of interest in the current study.

The review by Ranganathan and D’Souza (2006) found that the effects of THC on memory may vary according to the dose administered. Comparison of the effects of acute oral dosing with 7.5 mg of THC vs. 15 mg of THC found that THC impaired episodic memory and learning in a dose-dependent manner, with the larger dose resulting in greater detriment (Curran V. H. et al., 2002). Additionally, Abel (1971) found that the verbal recall of lists presented after THC administration was impaired, but the recall of lists presented prior to administration was not. Findings from animal models also suggest that THC changes behavior, impairs recognition, and impairs working memory in a dose-dependent manner (Stella, 2023; Verrico et al., 2012). Therefore, it is important to consider the THC dose when studying the effects of THC on memory. The ratio of THC to CBD varies widely among strains of legal-market cannabis (Curran T. et al., 2020; Pennypacker et al., 2022). High-potency THC strains with little to no CBD are on the rise, with the mean THC concentration of flower products increasing from 9.75% in 2009 to nearly 15% in 2018 (ElSohly et al., 2021). At the same time, some strains in Colorado test at greater than a 20:1 CBD to THC ratio, while others are closer to a 1:1 THC to CBD ratio.

Previous research is also limited by the use of cannabis strains that are unlike legal market cannabis products. Although cannabis is legal at the state level in Colorado, university researchers are not allowed to have participants use or handle state-legal cannabis in any form on university property or in the presence of university staff in compliance with the Drug-Free Schools Act. Therefore, researchers in conventional lab settings are confined to using NIDA-provided low-concentration THC products that lack CBD and use drug administration approaches that do not reflect the typical legal market use. Therefore, the findings from these studies may have limited generalizability to real-world cannabis use (Bidwell et al., 2018; Chait and Zacny, 1992; Schacht et al., 2009).

The current study extends the work of Curran T. et al. (2020), which used a mobile laboratory to address the above limitations and assess the acute effects of different THC/CBD ratios on memory via a verbal recognition task. This design facilitates the naturalistic administration of legal market cannabis that varies in THC and CBD ratios and the subsequent assessment of learning and memory outcomes after *ad libitum* use. Furthermore, based on preliminary results from Curran T. et al. (2020), we modified the mobile laboratory to host a verbal recognition memory task and a portable electroencephalography (EEG) unit to record participants’ brain waves as they completed the task within the mobile laboratory. Curran T. et al. (2020) was limited in that the design used both flower and concentrate forms of cannabis, varying potencies of products, and only two groups (e.g., a THC group and a THC + CBD group) to assess the effect of THC/CBD ratios on memory. The current study expands upon the previous design by limiting participant product use to legal market cannabis flower exclusively, standardizing product concentrations, and implementing three different groups (strains).

We hypothesized that THC would reduce recognition memory accuracy (i.e., resulting in smaller d’ values) and that when THC is combined with CBD, the CBD would reduce the effects of THC on memory. Thus, we predicted that d’ values would be lower under cannabis conditions for the +THC/-CBD strain compared to the -THC/+CBD and +THC/+CBD strains.

## Methods

In the current study, participants who reported regular cannabis use were randomly assigned to one of three strains of flower product with varying ratios of THC and CBD: a THC-based strain, an approximately 1:1 THC: CBD strain, or a CBD-enriched strain. Participants used the legal market products *ad libitum* in their private residences and then returned to the mobile laboratory for a series of assessments. We assessed the effects of each strain on recognition memory accuracy (d’), response bias (C), false alarm rate, hit rate, and reaction time by comparing the outcomes between a cannabis condition (i.e., an experimental session in which participants used legal market product) and a non-cannabis condition (i.e., an experimental session in which participants did not use any product). This design allows for self-dosing of the cannabis product, which may mimic the effects of real-world legal market product use.

### Participants

**Figure 1:**
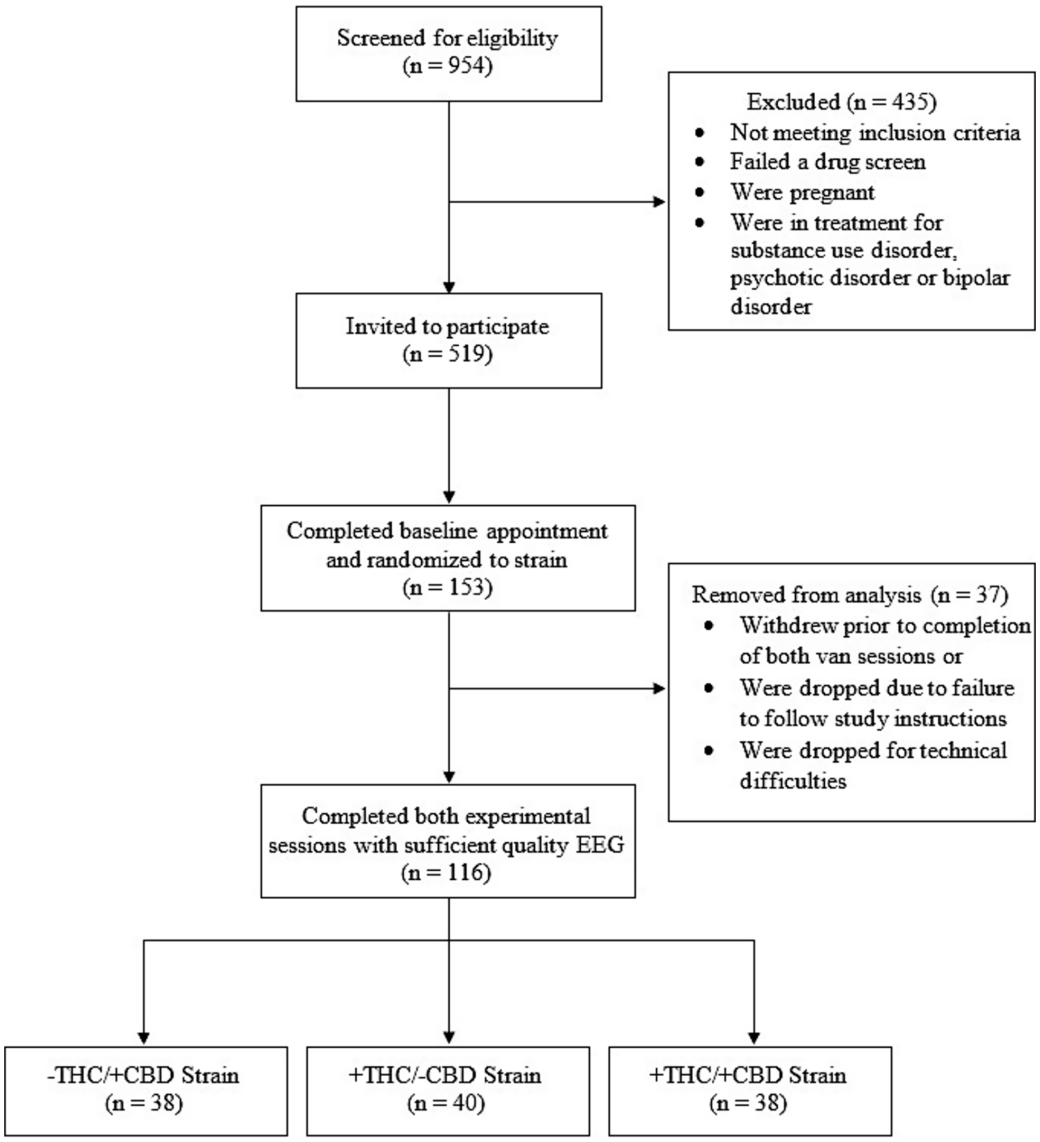
CONSORT diagram of participants (N).

Participants were recruited through flyers distributed at local dispensaries, Alesco mailed flyers^1^, tabling at local events, ResearchMatch^2^, and social media platforms. Participant inclusion criteria were that participants were predominantly right-handed (i.e., laterality quotient > 60 on the Edinburgh Handedness Inventory), between the ages of 21 and 40, had used cannabis at least 4 days during the previous month, had used cannabis for at least a year, had no other illicit recreational drug use (self-reported), were not using psychotropic medications (antidepressants, non-benzodiazepine anti-anxiety, and ADHD medications were allowed), and did not regularly use nicotine (≤4 days per week). Participants were randomly and evenly counterbalanced across session order, cannabis strain, and the old/new button condition. Refer to Figure 1 for participant (N) and Table 1 for participant demographics.

**Table 1:** Participant demographics by strain.

	Strain group			Sample total
	−THC/+CBD	+THC/−CBD	+THC/+CBD	
*N*	38	40	38	116
Age	28.53	27.10	27.34	27.65
Gender (#F)	21	18	19	58 (50%)
Race				
American Indian or Alaska Native	1 (2.63%)	0 (0.00%)	0 (0.00%)	1 (0.86%)
Asian	2 (5.26%)	2 (5.00%)	1 (2.63%)	5 (4.31%)
Black/African American	0 (0%)	2 (5.00%)	0 (0.00%)	2 (1.72%)
Native Hawaiian or Pacific Islander	0 (0%)	1 (2.50%)	1 (2.63%)	2 (1.72%)
White	29 (76.32)	29 (72.50%)	32 (84.21%)	90 (77.59%)
Multiple Races Reported	3 (7.90)	4 (10.00%)	3 (7.90%)	10 (8.62%)
Prefer Not to Respond	3 (7.90%)	2 (5.00%)	1 (2.63%)	6 (5.17%)
Ethnicity				
Hispanic/Latino	3 (7.90%)	10 (25.00%)	4 (10.53%)	17 (14.66%)
Not Hispanic/Latino	35 (92.11%)	29 (72.50%)	33 (86.84%)	97 (83.62%)
Prefer Not to Respond	0 (0.00%)	1 (2.50%)	1 (2.63%)	2 (1.72%)

The study was approved by the University of Colorado Boulder Institutional Review Board and followed all ethical standards and guidelines from the relevant national and institutional committees on human experimentation and the Helsinki Declaration.

#### Timeline and compensation

The total time commitment for participants was approximately 6 h, spread over 3 weeks. The first appointment, Baseline, averaged 90 min and occurred at the research lab. For the vast majority of participants, both experimental appointments (e.g., cannabis and non-cannabis van sessions) lasted about 2 h and occurred within 2 weeks of the Baseline appointment, with 2–7 days between van appointments. Participants were compensated $195 in cash for their participation in the study.

### Materials and equipment

#### Survey technology

Participants used an iPad to complete a variety of surveys at each appointment. All coded data were entered into the Research Electronic Data Capture (REDCap; Harris et al., 2009), a 21 CFR Part 11-ready data capture system provided by the University of Colorado Denver.

#### Mobile laboratory

On-site EEG recordings occurred in one of two nearly identical modified sprinter vans throughout the experiment. An EEG net recorded the participants’ brain electrical activity during the EEG tasks in the van. The van was selected for each participant based on availability, and we attempted to run the subjects in the same van for both sessions. 63.70% of appointments were run in Van 1, and 36.30% of appointments occurred in Van 2.

The cargo area of the vans was outfitted with lab equipment (blood caddy, EEG equipment, food and drink, and survey equipment) and heavy blinds to limit distractions, as well as a table, chair, monitor, and keyboard for participant use. An EGI (Electrical Geodesics, Inc.) EEG amplifier was also present on the desk but placed outside of the participants’ field of view. Two experimenters were present in the van throughout the experiment.

#### Experimental stimuli

Stimuli for the experimental task were words selected from the PEERS word pool (Aka et al., 2020). From these words, 28 lists (2 van sessions x 7 lists/van x 2 old/new conditions) were selected, each of which had 20 words that were roughly equated on concreteness, familiarity, and frequency, and were 4–8 letters in length. Each condition used the same lists for all subjects so that individual differences in the results would be less affected by item differences. Additional words (4–8 letters long) were selected for primacy and recency buffers in the study lists and practice/Baseline lists. All stimuli were presented via a computer monitor. Refer to the Supplementary material for details.

### Procedure

#### Baseline appointment

Participants who met the inclusion criteria were contacted to be scheduled for a Baseline appointment and were instructed not to use cannabis for 24 h preceding their Baseline appointment. Informed consent was obtained at the start of the Baseline appointment. Following completion of the consent form, a breathalyzer (Intoximeter, Inc., St. Louis, MO, United States) and urinalysis were administered to ensure that participants had no alcohol, sedatives, cocaine, opiates, or amphetamines in their system. Female participants were required to take a urine pregnancy test to ensure that they were not currently pregnant. Participants then provided a blood draw and completed the Baseline surveys. Data on age, sex, race, and ethnicity were collected for each participant. Participants also reported their substance use (including cannabis use) in the prior 30 days on the Timeline Follow-Back (Martin-Willett et al., 2020; Sobell and Sobell, 1992) as well as substance use history and age of first regular cannabis use. To prepare for the recognition and flanker tasks, participants completed a short familiarization task.

### Cannabis product assignment

Also at the Baseline appointment, participants were randomly assigned to one of three strains of cannabis: a THC-based strain akin to a typically used strain with normal levels of THC and little to no CBD (+THC/−CBD), a 1:1 strain with equivalent THC and CBD levels (+THC/+CBD), or a CBD-enriched strain with little to no THC (−THC/+CBD). The +THC/−CBD strain contained 12.5% THC and <1% CBD, the 1:1 +THC/+CBD strain contained 8.2% THC and 6.5% CBD, and the −THC/+CBD strain contained <1% THC and 17.4% CBD. The strains were selected to represent the range of THC and CBD potencies typically available in the legal market. Colorado requires all strains to be tested in a state-licensed laboratory. The THC and CBD potency of each study product was tested and labeled consistent with State of Colorado requirements in an International Organization for Standardization (ISO) 17025 accredited laboratory. ISO 17025 is the highest recognized quality standard in the world for the calibration and testing laboratories. Each participant was also given a card with directions to purchase their randomly-assigned study product in a pre-roll plant or flower form (to be smoked or vaporized) from a dispensary in Boulder, Colorado, United States. Participants were emailed a link after completion of the Baseline to upload a photo of their purchased product. Participants knew the THC/CBD concentration of their purchased strain, as the products were labeled for sale. Research staff instructed the participants to refrain from product use until their experimental session. See the Supplementary material for more details.

Researchers remained blind to which strain corresponded to which concentration, creating a single-blind experiment in which the research staff only knew whether participants were assigned to “Strain A,” “Strain B,” or “Strain C.”

#### Experimental appointments

Participants completed two separate experimental mobile laboratory appointments on two separate occasions (i.e., cannabis and non-cannabis van sessions). The van session order was counterbalanced among participants. The experimental appointments took place inside the mobile lab at a convenient location close to participants’ private residences (i.e., their driveway or a parking lot). The two experimental appointments were identical, except that one session (cannabis session) included participant self-administered in-home cannabis use (in accordance with state regulations) of the strain of cannabis assigned at the Baseline appointment. At the start of both van appointments, researchers first confirmed participant sobriety (e.g., caffeine, nicotine, alcohol, and ADHD medication free). Then, at both the cannabis and non-cannabis van sessions, and both pre-use and post-use in the cannabis session, participants completed both an intoxication scale (Bidwell et al., 2020) to self-report their degree of intoxication and blood draws. The intoxication scale consisted of three items (“Right now, I feel… [physically stoned, mentally stoned, high (as in ‘drug high’)]), which were scored on a 5-point Likert-type scale from “Not at all [0]” to “Extremely [4].” Blood draws were used to quantify peak participant THC and CBD blood levels.

At this point during the cannabis session, participants went to their private residence to use their cannabis product for 15 min *ad libitum*. Following use, participants returned to the mobile laboratory. Participant cannabis use was quantified by product weight before and after use via a researcher-provided gram scale. The amount of product used (in mg) was also used to roughly estimate the amount of each cannabinoid (THC and CBD) consumed based on the percentages of THC and CBD in each strain. Participants repeated the self-report intoxication scale and a blood draw to assess their blood-cannabinoid levels post-use.

At this point during the non-cannabis session, participants returned to their private residence to play Sudoku for 15 min. After participants returned to the van, they played Sudoku for another 12 min to approximate the time spent completing questionnaires in the cannabis session.

**Figure 2:**
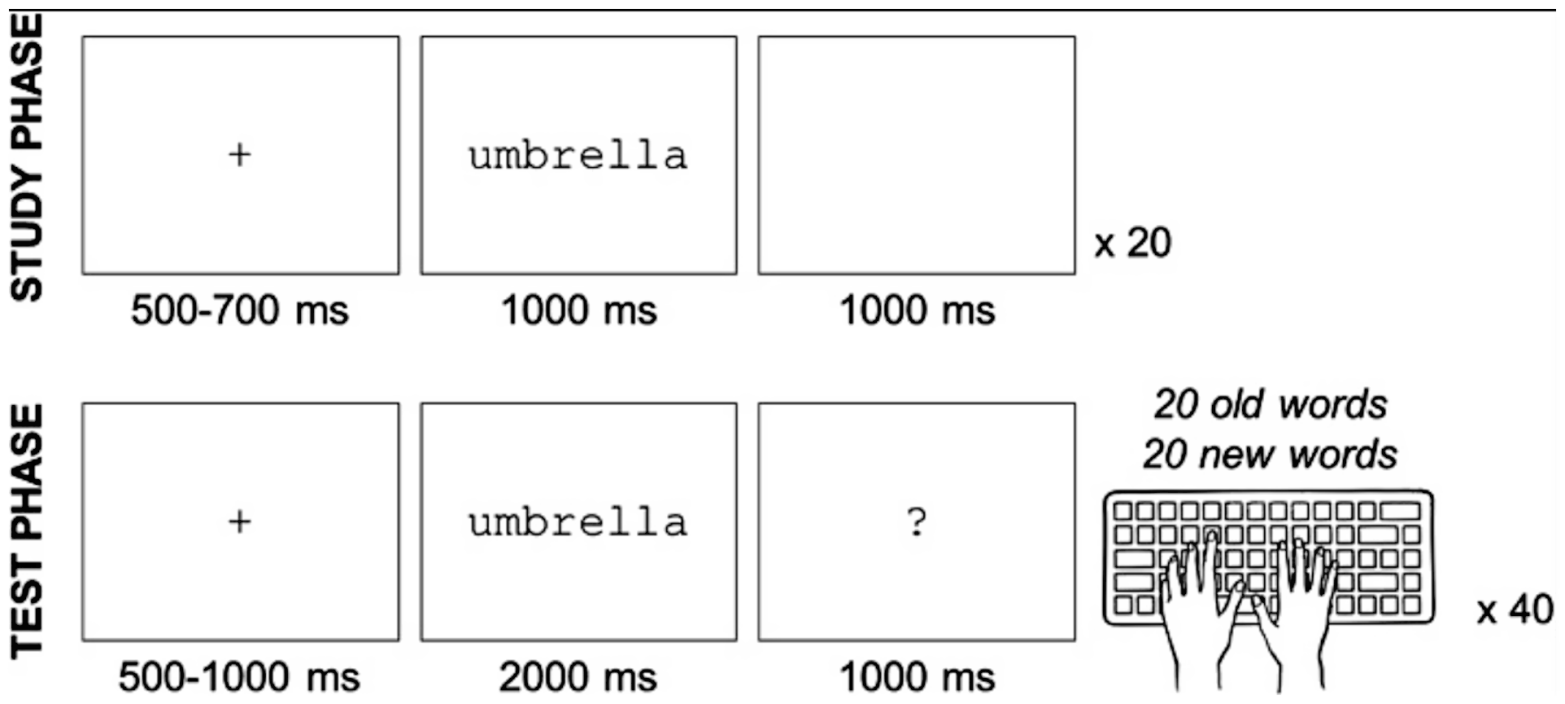
Overview of recognition memory task procedures. This figure is adapted from a preliminary study on memory recognition and cannabis use. See Curran et al. (2020) (Doi: 10.1186/s42238-020-00034-0).

Participants then completed the recognition memory task in both van sessions (see above and Supplementary material for task stimuli). The approximate time between the participants returning to the mobile laboratory and starting the recognition memory task was 12–15 min. Participants first studied a list of 20 words presented on a computer monitor. Following the study list, participants completed a recognition memory test with 40 words (20 old [studied] and 20 new [non-studied] words). Participants were instructed to judge each word as “old” (meaning they remembered seeing the word on the study list) or “new” (they did not remember seeing the word on the study list) by pressing either a leftward “F” key or a rightward “J” key on the keyboard (Figure 2). Assignment of response keys and left/right to old/new responses was counterbalanced across participants, and different words were used in cannabis/non-cannabis van sessions. There was a 3-min retention interval between each study and test list, during which participants completed a flanker task, determining the direction of rapidly appearing arrows on the screen. EEG was recorded while participants completed study lists, recognition, and flanker tasks. EEG and flanker task results will be published elsewhere.

### Statistical procedure

Experiment control was conducted using MATLAB (MATLAB, 2022) and the Psych Toolbox^3^ (Brainard, 1997; Pelli, 1997; Kleiner et al., 2007). All data cleaning and analyses were completed in R Statistical Software, version 4.2.1 (R Core Team, 2022). This was a repeated-measures design, given that participants completed both cannabis and non-cannabis van sessions. Mixed-methods repeated-measures analysis of variance (ANOVAs) were used to investigate strain differences in primary outcomes of interest. Unless otherwise stated, between-subjects variables were cannabis strain (−THC/+CBD, +THC/−CBD, or +THC/+CBD), sex of the participant (male or female), and order of the van session (cannabis session first or non-cannabis session first). The only within-subjects variable was the cannabis condition (cannabis vs. non-cannabis). Participant self-report of cannabis use frequency over the 30 days prior to the Baseline appointment was used as a covariate. Self-report of use frequency was chosen as a covariate as a measure of cannabis experience and tolerance, and because it was correlated more highly with our outcome measures than other potential covariates. Additional analyses controlled for intoxication effect in all models; results did not differ substantially from models that did not control for intoxication effects, so the simpler models were chosen and are presented in the Results. The simple effects of significant interactions were further investigated using estimated marginal means in order to ensure that estimates accounted for the full model, avoid inflating the Type I error rate, and decompose interactions into the levels of the factors. As such, the reported difference scores are for the estimated means rather than for the raw means. Refer to Table 2 for the raw means.

**Table 2:** Raw means by strain.

	Strain group		
	−THC/+CBD	+THC/−CBD	+THC/+CBD
Cannabis use			
First age of regular cannabis use	21.17	20.62	19.42
Cannabis days last 30 days (BL)	18.53	19.90	20.55
Acute use			
Product consumption (mg)	381.16	358.40	381.27
THC content (mg)	3.81	44.80	31.26
CBD content (mg)	66.32	3.58	24.78
D′			
D′ cannabis	1.99	1.57	2.07
D′ non-cannabis	1.87	1.96	2.12
Response bias			
C cannabis	0.21	−0.05	0.12
C non-cannabis	0.19	0.09	0.28
Reaction time			
RT cannabis	2.45	2.54	2.44
RT non-cannabis	2.46	2.42	2.42
False alarm rate			
False alarm cannabis	0.15	0.28	0.16
False alarm non-cannabis	0.18	0.19	0.13
Hit rate			
Hit rate cannabis	0.75	0.76	0.79
Hit rate non-cannabis	0.73	0.76	0.75

The cannabinoid content (THC and CBD) is a rough estimate of the amount of each cannabinoid consumed. This was calculated by multiplying the amount of product consumed by the percentages of THC and CBD contained within each product strain.

## Results

### Participants

A total of 116 participants were included in the analysis, with *N* = 38 in the −THC/+CBD strain, *N* = 40 in the +THC/−CBD strain, and *N* = 38 in the +THC/+CBD strain. The three groups did not differ significantly from one another in participant age at the time of the study, *F*(2, 113) = 0.61, *p* = 0.55; age at regular cannabis consumption, *F*(2, 109) = 1.63, *p* = 0.20; or Baseline assessment of participant cannabis use in the last 30 days, *F*(2, 113) = 0.49, *p* = 0.61. Finally, there was no significant mean difference among the strains in the average amount of product used during cannabis van sessions (*F*[2, 112] = 0.16, *p* = 0.85).

### Manipulation check

Investigation of the strain group mean differences in blood cannabinoid levels served as a manipulation check (see Table 3 for raw blood cannabinoid means). There were no significant mean differences in blood levels of THC, CBD, 11-OH-THC, or THC-COOH between strains at the Baseline appointment, the non-cannabis experimental session, or the pre-use timepoint within the cannabis experimental session (see Supplementary Figures 1–3). However, as expected for the post-use timepoint within the cannabis experimental session, THC levels were lower in participants in the −THC/+CBD strain than in the +THC/−CBD and +THC/+CBD strains (refer to Supplementary Table 1 for all difference statistics). There was no mean THC difference between participants in the +THC/−CBD strain and the +THC/+CBD strain, despite the difference in THC percentage between these two strains (12.5% vs. 8.2%). Also as expected, CBD levels were significantly lower in the +THC/-CBD strain compared to the −THC/+CBD and +THC/+CBD strains, with no significant mean difference in blood CBD levels between the −THC/+CBD and +THC/+CBD strains. Therefore, any difference in the results between the +THC/−CBD strain and the +THC/+CBD strain is likely due to the difference in CBD. Post-use blood levels of 11-OH-THC were lower in the −THC/+CBD strain than in the +THC/−CBD strain. There was no significant difference in the mean 11-OH-THC blood level between the −THC/+CBD strain and the +THC/+CBD strain, or between the +THC/−CBD strain and the +THC/+CBD strain. Participant blood THC-COOH levels did not differ between the strains. See Supplementary Figure 4 for more details on the strain differences in blood cannabinoids.

**Table 3:** Participant blood cannabinoid levels (ng/mL) by strain.

	Strain group		
	−THC/+CBD	+THC/−CBD	+THC/+CBD
Baseline appointment			
THC	8.84	6.30	11.82
CBD	6.53	0.73	1.92
THC-COOH	62.93	77.01	94.36
11-OH-THC	1.41	1.84	2.49
Non-cannabis experimental session			
THC	4.99	8.38	15.32
CBD	0.04	0.76	0.80
THC-COOH	52.85	86.30	130.58
11-OH-THC	0.90	1.85	4.01
Pre-use cannabis experimental session			
THC	6.36	12.60	10.64
CBD	0.07	0.46	0.11
THC-COOH	52.93	86.99	90.55
11-OH-THC	0.96	2.03	2.32
Post-use cannabis experimental session			
THC*	14.93	70.64	69.13
CBD*	38.20	0.43	21.79
THC-COOH	52.51	133.25	82.02
11-OH-THC*	2.35	9.03	5.67

* indicates that the effect of strain is significant on that cannabinoid.

Investigation of the self-reported intoxication effect (via the three-item intoxication scale) post-use in the cannabis experimental session also served as a manipulation check. As expected, given the manipulation of THC/CBD potency, participants in the −THC/+CBD group reported fewer intoxication effects than participants in both the +THC/−CBD strain (difference = 3.12, SE = 0.53, *p* < 0.001) and +THC/+CBD strain (difference = 3.29, SE = 0.54, *p* < 0.001). Intoxication effects did not differ between the +THC/−CBD and +THC/+CBD strains, which was expected considering that CBD is non-intoxicating and that blood levels of THC did not differ between these strains. Therefore, strain manipulation was effective in altering both participant blood cannabinoid levels and intoxication effects in the intended directions.

### Primary analyses

**Figure 3:**
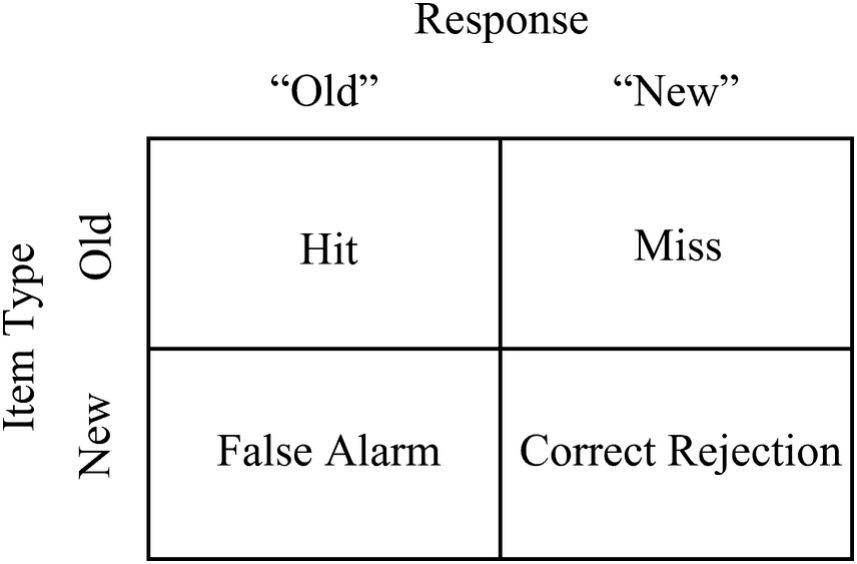
Response categorization of verbal recognition task stimuli.

Our primary measure of interest is accuracy in discriminating old vs. new words, or d’, in a recognition memory task. The hit rate (H, proportion of correct “old” responses to previously studied words) and the false alarm rate (FA, the proportion of incorrect “old” responses to non-studied words) are used to calculate d’ (d’ = *Z*_H_ – *Z*_FA_, where *Z* is the standard normal distribution). Hit and FA rates are also interesting on their own for assessing performance on old vs. new items. See Figure 3 for the response categorizations. We were also interested in response bias, or C (C = −1/2 * [*Z*_H_ – *Z*_FA_]). A zero value for response bias indicates that participants responded with half “old” and half “new” responses. A negative response bias value indicates a more liberal response, such that participants respond “old” more often than “new.” Conversely, a positive response bias value indicates a more conservative response, such that participants respond “new” more often than “old.” Generally, a more liberal response bias results in more hits and false alarms, whereas a conservative response bias results in fewer hits and false alarms. Although d’ is intended to measure accuracy in a way that is uninfluenced by response bias, more recent work has suggested that d’ is somewhat influenced by response bias, which is another reason to consider the effects of response bias (Brady et al., 2023).

#### Accuracy (d’)

**Figure 4:**
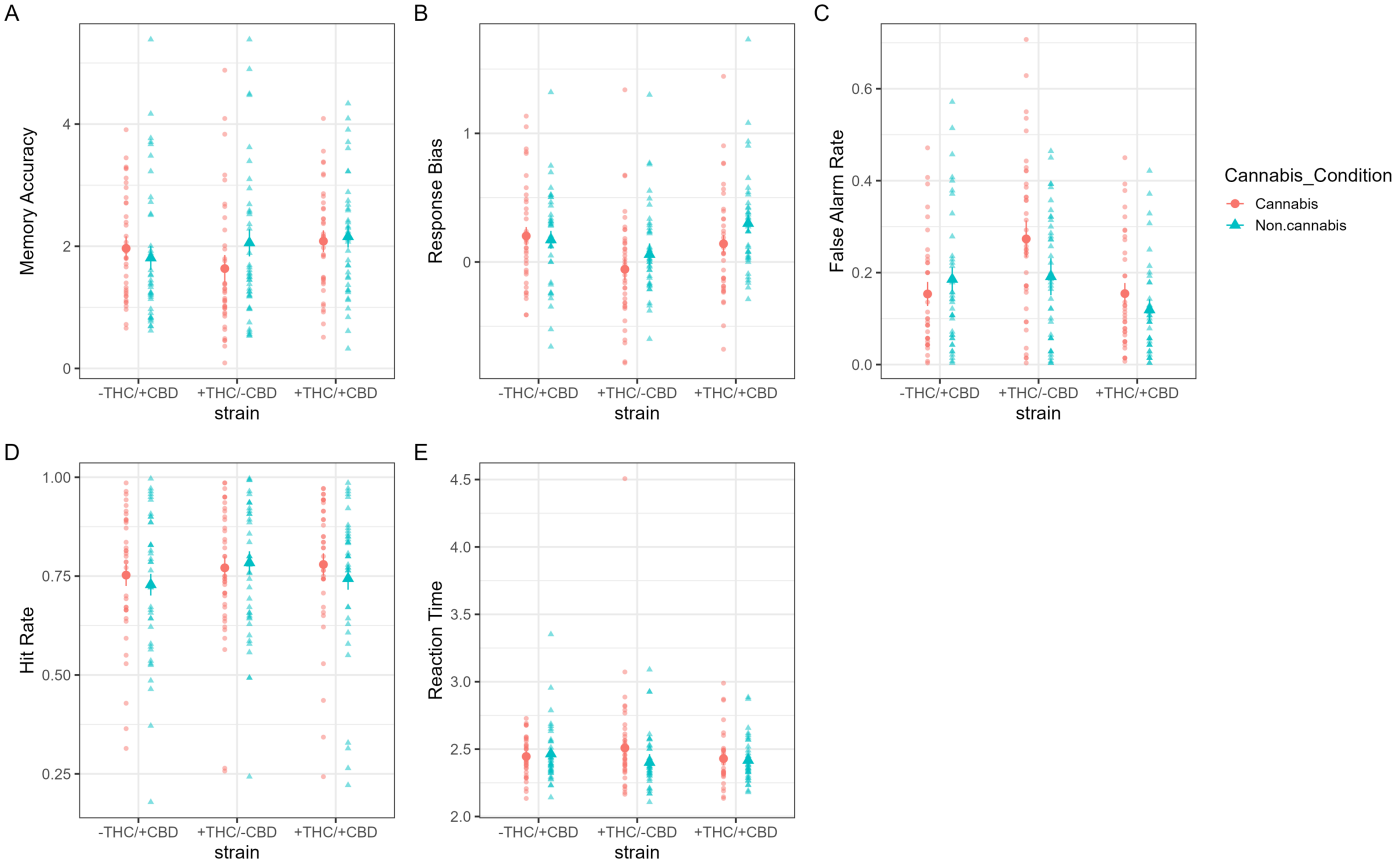
**(A–E)** From left (top) to right (bottom): **(A)** memory accuracy (*d*’, dprime), **(B)** response bias, **(C)** false-alarm rate, **(D)** hit rate, and **(E)** reaction time under strain and cannabis conditions. Large shapes are means with standard errors. Small shapes represent individual participants. Error bars show within-subject standard errors.

The results of the mixed-methods repeated-measures ANOVA are shown in Figure 4A. The ANOVA yielded a significant two-way interaction between strain and cannabis condition [*F*(2, 103) = 4.70, *p* = 0.01, partial $\eta^{2}$ = 0.08]. Specifically, the investigation of simple effects revealed that the effect of the cannabis condition was only significant for the +THC/−CBD strain, such that d’ for the cannabis condition was lower than d’ for the non-cannabis condition (difference = 0.42, SE = 0.134, *p* = 0.002). The effect of cannabis condition was not significant in either the −THC/+CBD or +THC/+CBD strains. Investigation of the two-way interaction between strain and cannabis condition was the primary aim of the current study; refer to the Supplementary material for additional results.

#### Response bias (C)

The results of the mixed-methods repeated-measures ANOVA are shown in Figure 4B. The analysis revealed a significant two-way interaction between strain and cannabis condition [*F*(2, 103) = 3.82, *p* = 0.025, partial $\eta^{2}$ = 0.07]. Investigation of simple effects revealed that the effect of cannabis condition on response bias was not significant for the −THC/+CBD strain. However, cannabis condition had similar effects on the other two strains, where the cannabis condition led to more liberal response bias than the non-cannabis condition (+THC/−CBD: difference = 0.12, SE = 0.05, *p* = 0.02; +THC/+CBD: difference = 0.15, SE = 0.05, *p* = 0.002). Refer to the Supplementary material for additional results.

#### False alarm rate

The results of the mixed-methods repeated-measures ANOVA are shown in Figure 4C. The analysis yielded a significant two-way interaction between the strain and cannabis condition [*F*(2, 103) = 7.71, *p* < 0.001, partial $\eta^{2}\;$ = 0.13], such that the effect of cannabis condition on false alarm rate is only significant for the +THC/−CBD strain, with a greater false alarm rate in the cannabis condition than in the non-cannabis condition (difference = 0.08, SE = 0.02, *p* < 0.001). Refer to the Supplementary material for additional results.

#### Hit rate

The results of the mixed-methods repeated-measures ANOVA are shown in Figure 4D. The analysis revealed that the primary interaction of interest, the two-way interaction between strain and cannabis condition, was not significant for hit rate. Refer to the Supplementary material for additional results.

#### Reaction time (RT)

A mixed-methods repeated-measures analysis of variance (ANOVA) was used to investigate group differences in reaction time (Figure 4E). The analysis used strain, sex of the participant, and order of the van session as between-subjects variables and cannabis condition, old vs. new words, and correct vs. incorrect response as within-subjects variables, and used participant self-report of cannabis use frequency as a covariate. The two-way interaction between strain and cannabis condition was significant [*F*(2, 97) = 3.25, *p* = 0.04, partial $\eta^{2}\;$ = 0.06], with the effect of cannabis on reaction time significant only for the +THC/-CBD strain, with the average reaction time being slower for the cannabis condition than for the non-cannabis condition (difference = 0.11, SE = 0.04, *p* = 0.004). Analyses were also conducted without the extreme reaction time outlier in the +THC/−CBD strain (see Figure 4E); analyses did not substantially change without the outlier, so the outlier was left in the analysis. Refer to the Supplementary material for additional results.

### Free recall results

**Figure 5:**
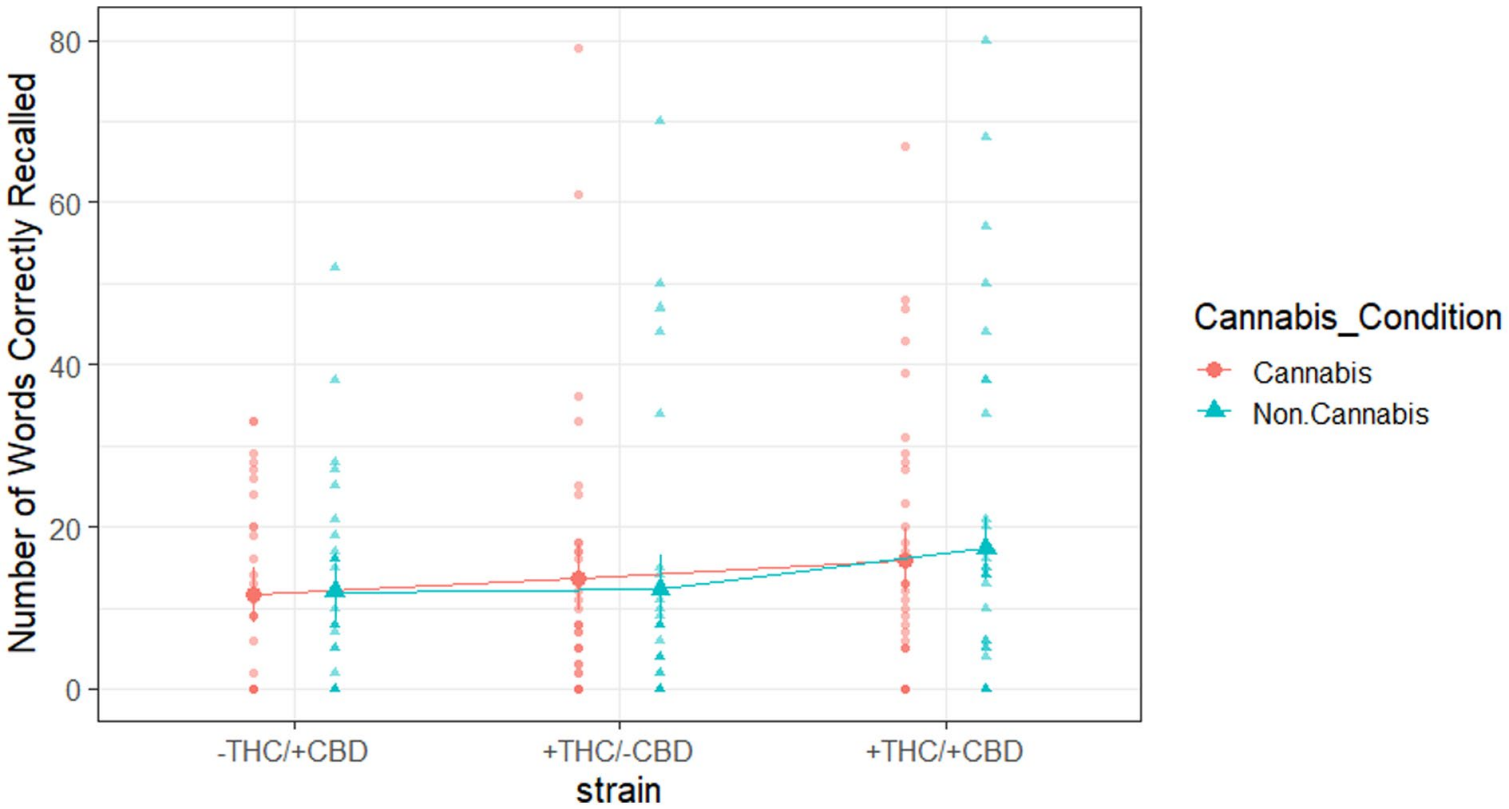
Words correctly recalled from any of the seven study lists, broken down by strain and cannabis condition. Large shapes are means with standard errors. Small shapes are individual participants. Error bars show within-subjects standard error.

We were secondarily interested in potential strain-by-cannabis condition effects on the recall task. Specifically, a mixed-methods repeated-measures ANOVA on the number of words correctly recalled from any of the seven study lists used strain as a between-subjects variable and the cannabis condition as a within-subjects variable. The analysis yielded null results (Figure 5), indicating that the interaction between strain and cannabis condition was not significant [*F*(2, 97) = 0.30, *p* = 0.75, partial $\eta^{2}$ = 0.01] for the number of words correctly recalled.

## Discussion

Our hypothesis that THC will result in lower recognition memory accuracy and that when THC is combined with CBD, the CBD may reduce the effects of THC on memory, was supported. Analyses revealed significant strain * cannabis condition effects for d’ and false alarm rate only for the +THC/−CBD strain, such that d’ was lower in the cannabis condition and the false alarm rate was higher in the cannabis condition. Analyses also found a significant strain * cannabis condition effect for response bias for the +THC/−CBD strain, with a more liberal response bias in the cannabis condition. The lack of significant cannabis effects on memory (d’) in the +THC/+CBD strain suggests a protective effect of CBD when combined with THC. Furthermore, the significant two-way interaction between strain and cannabis condition for the +THC/−CBD strain on d’ should be interpreted in the context of a liberal response bias, such that participants tended to respond “old” more often than “new” in response to presented stimuli during the test tasks. This response bias effect may have influenced the d’ results, but if the +THC/−CBD strain only affected the response bias, we should have observed that the cannabis condition increased the rate of both false alarms and hits, but no such effect was observed for hits. Notably, the effect of cannabis condition was significant for response bias in both the +THC/−CBD and +THC/+CBD strains, whereas the effect of cannabis condition was only significant for d’ in the +THC/−CBD strain. The greater false alarm rate for participants using the +THC/−CBD strain, which is consistent with the findings of Ilhan et al. (2004) and Abel (1971), suggests that the observed effects may be specific to THC impacting the perceived memorability of new items. The reaction time analysis also confirmed that +THC/−CBD use impaired performance by slowing the reaction time, but as for the accuracy-related measures above, that effect was also not observed when higher levels of CBD were combined with THC (+THC/+CBD).

We also predicted that a primarily CBD strain would have no effect on memory. This hypothesis was also supported and is in line with previous studies (Gebregzi et al., 2025), as there was no significant effect of cannabis condition on memory accuracy (d’), response bias, false alarm rate, hit rate, or reaction time for the -THC/+CBD strain. Although the estimated mean difference in d’ between the non-cannabis and cannabis conditions was not significant (difference = 0.16, SE = 0.14, *p* = 0.25), the estimated mean d’ for the cannabis condition (*M* = 1.96) trended higher than the estimated mean d’ for the non-cannabis condition (*M* = 1.81). This trend should be a subject of future studies, as it may suggest that CBD attenuates THC-related impairment on memory and may improve accuracy, which is consistent with previous animal studies (Boggs et al., 2018; Wright et al., 2013).

Our analysis of the strain-by-cannabis condition effects on the number of words correctly recalled from any of the seven study lists yielded null results. One possible reason for this could be that subjects were no longer intoxicated when they completed the recall task 1 h and 51 min after using their product. The results of our recognition task analyses suggest that THC impaired recognition memory, but when the strain included both CBD and THC, CBD counteracted the effects of THC alone. These effects could have influenced memory encoding processes during study, memory retrieval processes during test, or both. If the primary effects were on memory encoding processes, we should expect them to also influence later recall results, even if the subjects are no longer intoxicated during the recall task. Since we observed no such effects in the recall results, this may suggest that the effects on recognition memory performance primarily influenced memory retrieval processes. During the recognition blocks, subjects were still intoxicated, so memory retrieval processes were affected; however, during recall, subjects were sober, so the retrieval processes were not affected. Future studies should investigate the effect of cannabis use on retrieval processes alone.

The manipulation of strain was effective. Blood cannabinoid levels matched expectations based on strain, with the effect of strain on blood cannabinoid levels being significant at the post-use cannabis condition experimental session for THC, CBD, and 11-OH-THC. Participants using the -THC/+CBD strain had lower levels of THC compared to participants using the +THC/−CBD and +THC/+CBD strains, and participants using the +THC/−CBD strain had lower levels of CBD than those using the −THC/+CBD and +THC/+CBD strains. Additionally, participants in the -THC/+CBD strain reported fewer cannabis effect sensations than participants in the +THC/−CBD and +THC/+CBD strains. Importantly, participant blood THC levels did not differ between the +THC/−CBD and +THC/+CBD strains; therefore, the difference in results between these strains was due to the difference in CBD between strains.

Our results differ from those of Englund et al. (2023), who found that while THC was associated with impaired delayed verbal recall, CBD did not modulate the effect of THC on recall. However, we found that not only does THC impair performance on a recognition task, but also CBD may reduce the impairing effects of THC on recognition. The difference in CBD results between the studies is likely due to several methodological differences. First, our experimental sessions focused on a verbal recognition task, whereas Englund et al. (2023) used a verbal recall task. The use of different tasks could have contributed to these different findings. Next, participants in the current study are more experienced with cannabis (*M* = 19.59 use days in the last 30 days) than those in Englund et al. (2023) (*M* = 4 use occasions in the last year). Previous studies have suggested that frequent cannabis users may experience different memory effects than those who do not use as frequently (Morgan et al., 2010; Morgan et al., 2018). In addition, a critical difference is that we employed a naturalistic design in which participants used legal market products *ad libitum*, whereas Englund et al. (2023) assigned all participants equivalent amounts of granulated cannabis vapor. Consistent with our naturalistic design—where individuals who regularly use legal market flower were allowed to self-titrate to their desired level of cannabis use—THC exposure in the current study appears greater than in Englund et al. (2023), as shown by participant blood levels. Our results also differ from those of Morgan et al. (2018). Given the methodological similarities between Englund et al. (2023) and Morgan et al. (2018) (e.g., use of vaporized cannabis, examination of a different memory outcome compared to the current study), the difference in results is likely due to methodological differences between Morgan et al. (2018) and the current study. Studies that allow more naturalistic dosing and administration procedures are critical complements to controlled dosing designs when addressing questions regarding cannabis impairment and effects. In addition, whether CBD interacts differently with THC at varying levels of THC exposure is an important question for future research.

The current study’s naturalistic design allowed for self-dosing of the cannabis product, which is a unique strength of this study, as it may mimic the effects of real-world legal market product use. The finding that THC lowered recognition memory accuracy has potential implications for public health, as does the finding that CBD may reduce the impairing effects of THC on memory. Individuals using primarily THC-based products may consider using strains that also incorporate CBD in order to help offset the potential reduction in recognition memory, especially as there was no significant difference in bodily sensations of intoxication effects between the +THC/−CBD and +THC/+CBD strains. However, our study has some limitations. Although researchers involved in data collection were blinded to participants’ cannabis strain assignments, due to the use of legal market products, participants were aware of the THC and CBD concentrations in their assigned product. Consequently, this study employed a single-blind rather than a double-blind design. It is important to note that the current study was limited to investigating acute effects on verbal recognition memory. Future research should investigate the potential effects of THC on non-verbal memory tasks. Furthermore, the current study used cannabis flower exclusively. Future analyses should consider the potential effects of other forms of cannabis products, such as edibles or concentrates, on recognition tasks and recall.

## Conclusion

The current study found evidence to suggest that cannabis flower products that are predominantly THC may contribute to less accurate recognition memory and slower reaction time. The finding that CBD may reduce the effects of THC on memory might have important implications for the use of CBD to counteract the detrimental recognition memory effects of THC, although more research is needed on this effect, given the mixed results of previous studies. Future research is needed to better understand the potential effects of THC and CBD on other forms of memory recognition and recall.

## Data Availability

Data analyzed for the present study are available upon request from the corresponding author due to the sensitive nature of the data (substance use).

## Footnotes

^1^ https://alescodata.com

^2^ https://www.researchmatch.org/

^3^ http://psychtoolbox.org/
