# Supplementary material for: Naturalistic investigation of cannabis strains varying in THC and CBD ratios and verbal recognition memory
Source: Front Psychol. 2026 Jan 6;16:1685412. doi: 10.3389/fpsyg.2025.1685412 (PMC12816388; doi:10.3389/fpsyg.2025.1685412)
Supplement: Supplementary file 1 [file Table_1.DOCX]

**Supplementary File**

Katie N. Paulich^1^, Christian Place^1^, Gregory Giordano^2^, William B. Carpenter^2,3^, Tim Curran^2*^, L. Cinnamon Bidwell^1,2*^

**eMethods 1.** Electrophysiological measures

**eMethods 2.** Baseline appointment procedure

**eMethods 3.** Experimental appointment procedure

**eMethods 4.** Statistical procedure

**eTable 1.** Post-use blood cannabinoids by strain

**eFigure 1.** Mean blood cannabinoid levels by strain at the baseline appointment

**eFigure 2.** Mean blood cannabinoid levels by strain at the non-cannabis experimental appointment

**eFigure 3.** Mean blood cannabinoid levels by strain at pre-use during the cannabis experimental appointment.

**eFigure 4.** Mean blood cannabinoid levels by strain at post-use during the cannabis experimental appointment

**eResults 1.** Additional blood analyses

**eResults 2**. Accuracy (d’) additional results

**eFigure 5.** Significant two-way interaction between the number of cannabis use days reported at the baseline appointment (BL Days) and cannabis condition

**eResults 3**. Response bias (C) additional results

**eResults 4.** False alarm rate additional results

**eResults 5.** Hit rate additional results

**eResults 6.** Reaction time (RT) additional results

**eMethods 1.** Electrophysiological Measures

An EEG net recorded participants’ brain electrical activity during the EEG tasks in the van. EEG was recorded continuously from 128 silver/silver chloride electrode scalp locations using a HydroCel Geodesic Sensor Net (Tucker, 1993) connected to a DC-coupled, 128-channel, high-input impedance amplifier (200 MΩ, NetAmps 400, Electrical Geodesics, Inc., Eugene, OR). Faraday cloth was used to minimize participant EMF exposure. The analog EEG signal was then converted to a digital array that was stored on a Macintosh computer. The EEG was referenced to the vertex electrode, band-pass filtered (0.1–100 Hz), and digitized at a sampling rate of 250 Hz. Electrode impedances were kept below 50 kΩ, an acceptable range for this system.

Stimuli for the experimental task were words selected from the PEERS word pool. The MRC Psycholinguistic Database was used to select words that were relatively concrete (MRC concreteness rating 400 – 670), highly familiar (MRC familiarity rating 308-646), mid-range in frequency (MRC Kucera-Francis written frequency 10-967), and 4 to 8 letters in length. From these words, 28 lists were selected that each had 20 words that were roughly equated on concreteness (range of 28 means within each list: 511 to 563), familiarity (range of 28 means: 495 to 540), frequency (range of 28 means: 23.9 to 24.2), and length (range of 28 means: 5.0 to 6.1). Twenty-eight was the total number of lists needed per subject: 2 van sessions (intoxicated vs. non-intoxicated) x 7 lists/van x 2 old/new conditions. Rather than counterbalancing lists across conditions, each condition used the same lists for all subjects so that individual differences in results would be less affected by item differences. Additional words were selected for primacy and recency buffers in study lists (14 lists x 6 buffers/list = 84) and practice/baseline lists (26 study words + 10 new test words = 36). These buffers and practice words were not controlled for concreteness, familiarity, or frequency statistics, but were all 4-8 letters long.

All stimuli were presented at the center of a 17-inch LCD computer monitor with a display resolution of 1,280 × 1,024 pixels and refresh rate of 60 Hz. Words were presented in Arial font, with a font size of 30. The screen was 50cm away from the participants face during the experiment, presenting words with a horizontal visual angle between 4° and 6°, varying by word length.

**eMethods 2.** Baseline Appointment Procedure

Participants who met inclusion criteria were contacted to be scheduled for a Baseline appointment, and were instructed not to use cannabis for 24 hours preceding their Baseline appointment. Informed consent was obtained at the start of the Baseline appointment.

Following completion of the consent form, a breathalyzer (Intoximeter, Inc., St. Louis, MO) and urinalysis test was administered to ensure that participants had no alcohol, sedatives, cocaine, opiates, or amphetamines in their system. If either test was positive, the Baseline appointment was rescheduled. Participants with repeated positives were terminated from the study. Female participants were required to take a urine pregnancy test to ensure that they were not currently pregnant. Participants then provided a blood draw and physiological measures, and then filled out the battery of Baseline surveys (see above) on an iPad. After the surveys, participants completed a short familiarization task to prepare for the van appointments. The familiarization task included the tasks used in the van sessions: A 26-word study list, followed by 3 minutes of the flanker tasks, followed by a 20-word recognition test list. Before leaving the baseline appointment, each participant was given a card with directions to a local dispensary in order to purchase their randomly-assigned study product. Participants were emailed a link after completion of the Baseline to upload a photo of their purchased product. To maintain the blinding, a member of the study team not involved in data collection reviewed the product photos to verify the participant correctly purchased the assigned strain and to record product name and cannabinoid potencies.

**eMethods 3*.*** Experimental Appointments Procedure

Following the cannabis and non-cannabis experimental conditions, participants then completed the recognition memory task in both van sessions. First, participants studied a list of 20 words presented on the computer monitor. The study lists also included three, non-tested buffer items at the beginning and end of the list to reduce primacy and recency effects. Following the study list, participants completed a recognition memory test with 40 words; containing 20 old (studied) and 20 new (non-studied) words. Fixation crosses were randomly timed between 500-700 ms for study trials and between 500-1000ms for test trials. Participants were instructed to judge each word as “old” (meaning they remembered seeing the word on the study list) or “new” (they did not remember seeing the word on the study list) by pressing either a leftward “F” key or a rightward “J” key on the keyboard. If the word was “old” and the participant responded “old,” it is considered to be a hit. If the word was “old” and the participant responded “new,” it is considered a miss. If the word was “new,” but participants responded “old,” that would be considered a false alarm. Finally, if the word was “new,” and participants responded with “new,” it is considered a “correct rejection.” Participants were required to wait for the “?” before responding on test trials. This was done to remove response related activity from the EEG. Although reaction time (RT) analyses are presented below, this delayed response procedure could have made RT effect less likely to be observed. Assignment of response keys and left/right to old/new responses was counterbalanced across participants, and different words were used in intoxicated/non-intoxicated van sessions. There was a three-minute retention interval between each study and test list, during which participants completed a flanker task, determining the direction of rapidly-appearing arrows on the screen. EEG was recorded while participants completed study lists, recognition, and flanker tasks. EEG and flanker task results will be published elsewhere.

**eMethods 4.** Statistical Procedure

Participants completed a total of seven blocks for ERP analyses, with one task stimuli list presented per block. However, preliminary analyses on our outcomes of interest using strain, participant sex, and order of van session as between-subjects variables and cannabis condition and block as within-subjects variables suggested that there was no significant effect of block on analyses or on participant fatigue, so data were aggregated across block for analyses. Results presented in the main text are for data aggregated across block.

| **eTable 1.** Post-Use Blood Cannabinoids Difference Statistics by Strain | | | | |
| --- | --- | --- | --- | --- |
| **Cannabinoid** | **Comparison** | **Difference** | **SE** | **p-value** |
| THC* |  |  |  |  |
|  | -THC/+CBD : +THC/-CBD | -55.72 | 16.30 | 0.003 |
|  | -THC/+CBD : +THC/+CBD | -54.20 | 16.50 | 0.004 |
|  | +THC/-CBD : +THC/+CBD | 1.51 | 16.50 | 0.995 |
| CBD* |  |  |  |  |
|  | -THC/+CBD : +THC/-CBD | 37.80 | 7.27 | <.001 |
|  | -THC/+CBD : +THC/+CBD | 16.40 | 7.32 | 0.07 |
|  | +THC/-CBD : +THC/+CBD | -21.40 | 7.32 | 0.01 |
| 11-OH-THC* |  |  |  |  |
|  | -THC/+CBD : +THC/-CBD | -6.68 | 2.03 | 0.004 |
|  | -THC/+CBD : +THC/+CBD | -3.31 | 2.05 | 0.24 |
|  | +THC/-CBD : +THC/+CBD | 3.37 | 2.05 | 0.23 |
| THC-COOH |  |  |  |  |
|  | -THC/+CBD : +THC/-CBD | -80.70 | 33.70 | 0.05 |
|  | -THC/+CBD : +THC/+CBD | -29.50 | 34.00 | 0.66 |
|  | +THC/-CBD : +THC/+CBD | 51.20 | 34.00 | 0.29 |

*Note*. * indicates the effect of strain was significant on that cannabinoid. For THC, *F*(2, 101) = 7.51, *p<*.001). For CBD, (*F*(2, 101) = 13.59, *p*<.001), and for 11-OH-THC, (*F*(2,101) = 5.39, *p* = .01), and for THC-COOH (*F*(2 , 101) = 2.93, *p* = .06). A negative difference means that lower levels of that cannabinoid were found in the first strain in the comparison. For example, levels of THC were significantly lower in the -THC/+CBD strain compared to the +THC/-CBD strain.

**
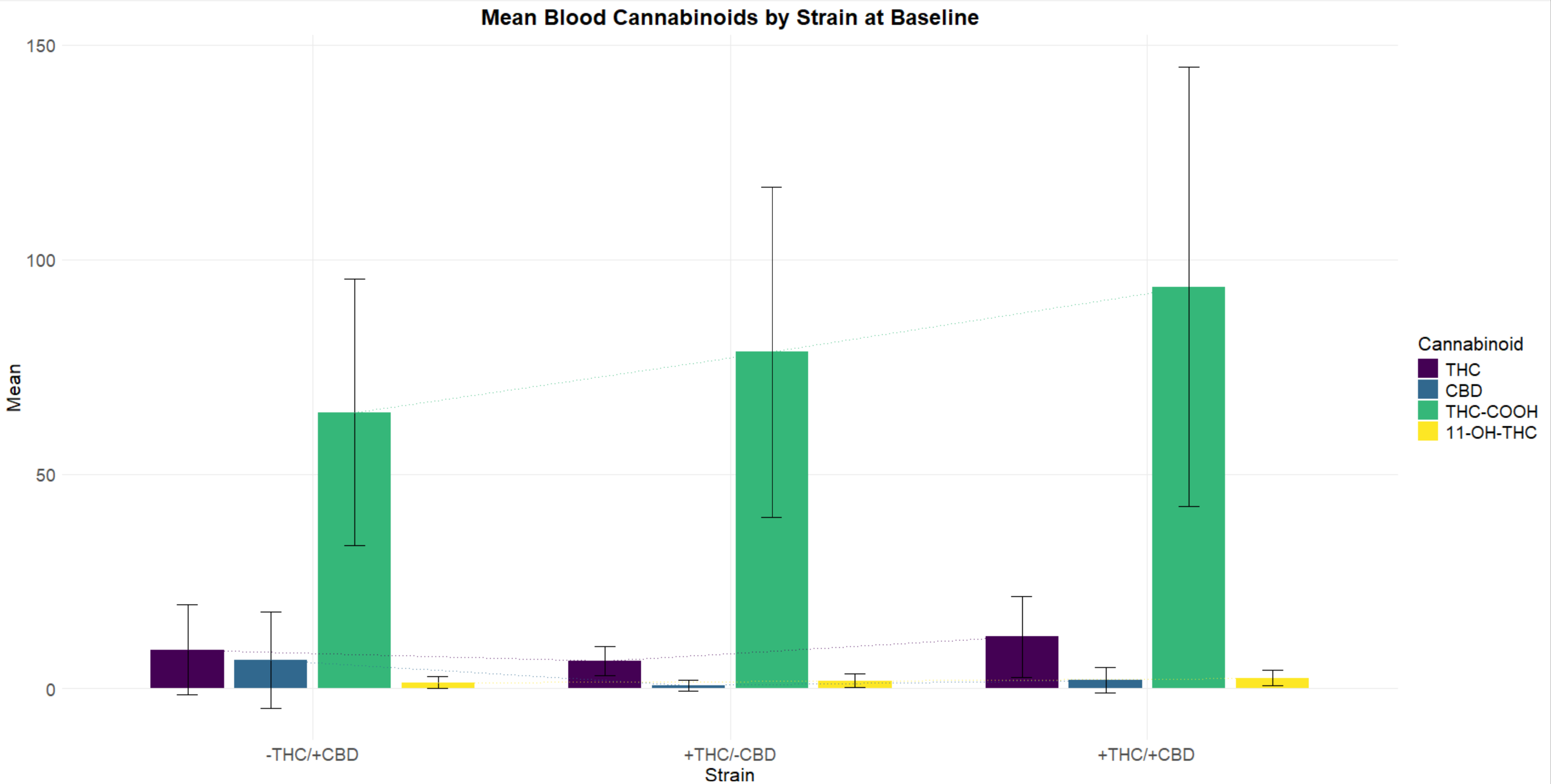
**

**eFigure 1.** Mean blood cannabinoid levels by strain at the baseline appointment. There were no significant differences in any cannabinoids among the strains.

**
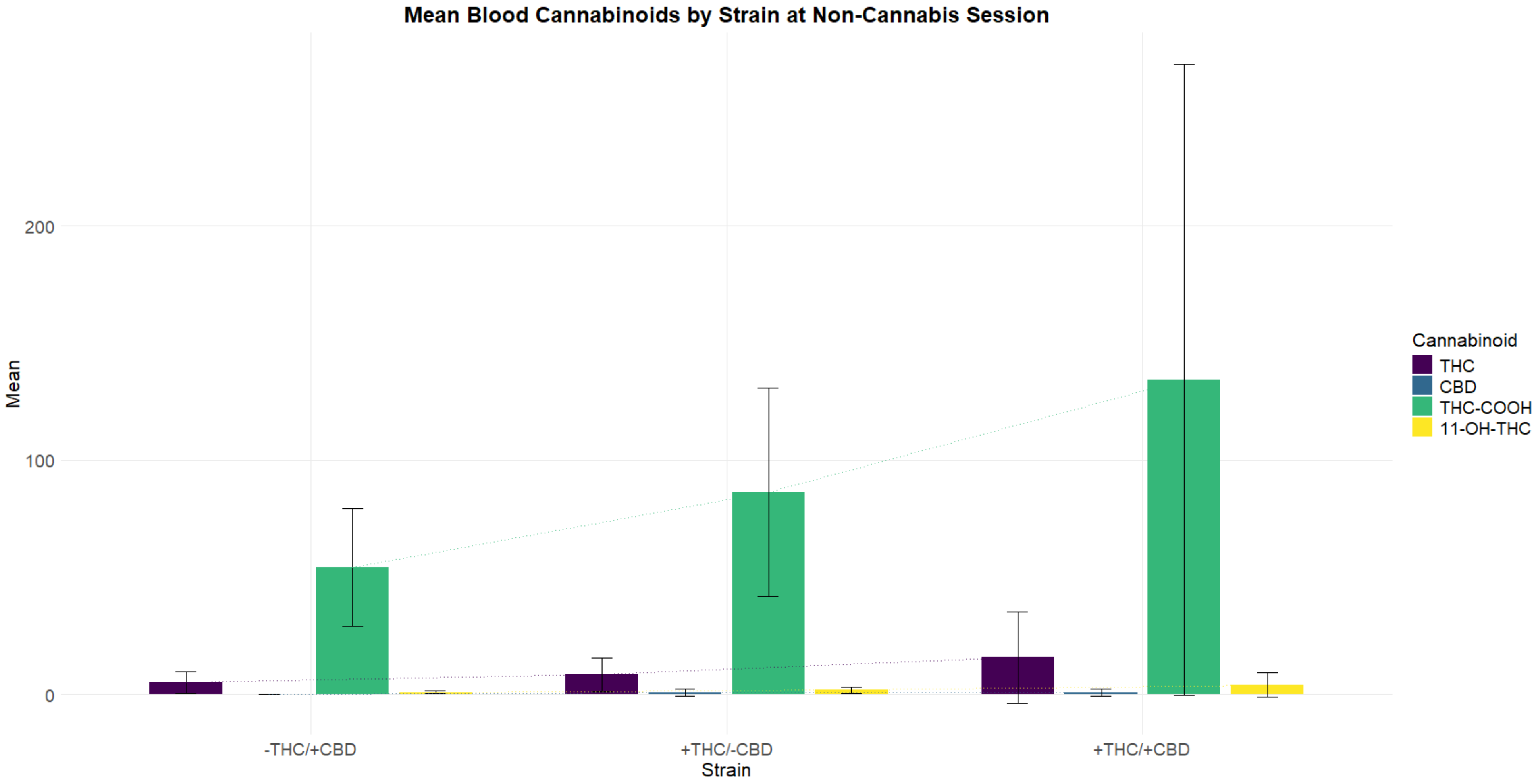
**

**eFigure 2.** Mean blood cannabinoid levels by strain at the non-cannabis experimental appointment. There were no significant differences in any cannabinoids among the strains.

**
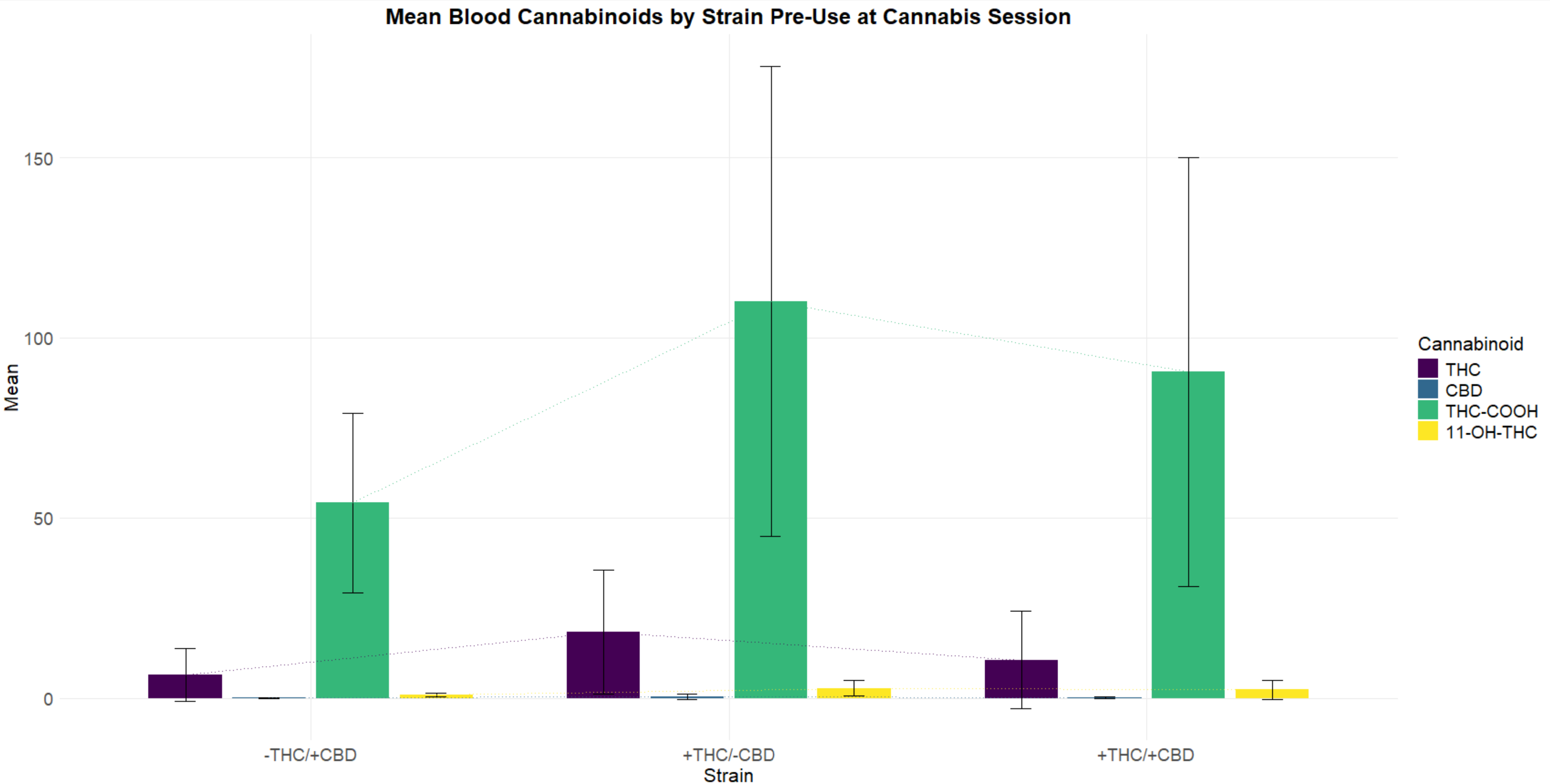
**

**eFigure 3.** Mean blood cannabinoid levels by strain at pre-use during the cannabis experimental appointment. There were no significant differences in any cannabinoids among the strains.

**
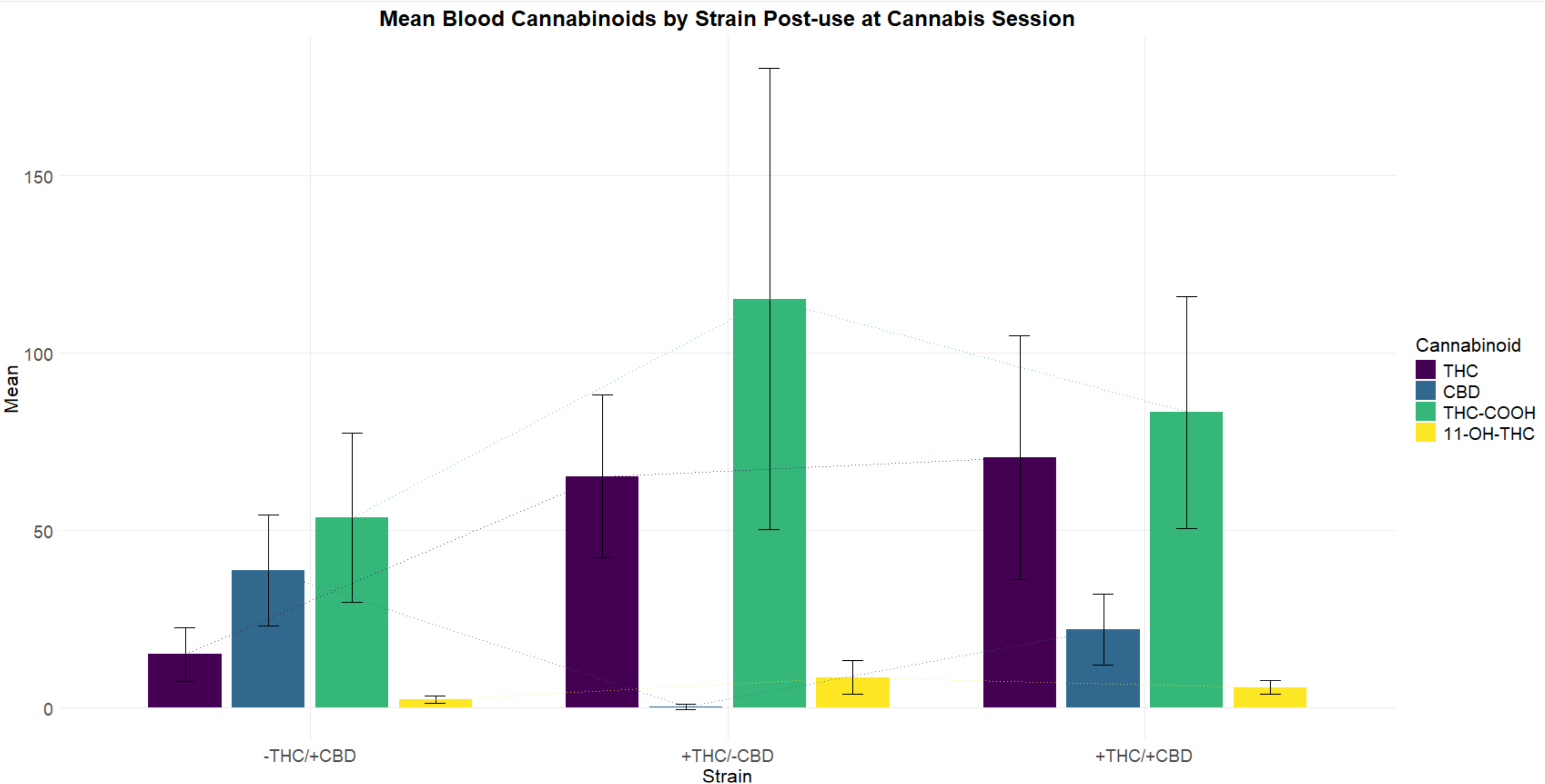
**

**eFigure 4.** Mean blood cannabinoid levels by strain at post-use during the cannabis experimental appointment. See eTable 1 for difference statistics and significance.

**eResults 1.** Additional Blood Analyses

Participant blood cannabinoid levels (i.e., THC, CBD, THC-COOH, and 11-OH-THC), obtained via blood draw at the Baseline appointment, non-intoxicated experimental session, and both pre- and post-use at the intoxicated experimental session, were also of interest. Differences in pre- and post-use blood cannabinoid levels were assessed. For the -THC/+CBD strain, post-use blood cannabinoid levels were significantly higher than pre-use levels for THC (*t*(34) = -3.01, *p* = .005); CBD (*t*(34) = -5.07, *p*<.001); and 11-OH-THC (*t*(34) = -3.45, *p* = .002), but there was no significant difference in THC-COOH. For the +THC/-CBD strain, post-use blood cannabinoid levels were significantly higher than pre-use levels for THC (*t*(32) = -4.50, *p*<.001); 11-OH-THC (*t*(32) = -3.33, *p* = .002); and THC-COOH (*t*(32) = -2.96, *p* = .006, but there was no significant difference in CBD levels (*t*(32) = 0.95, *p* = .35). For the +THC/+CBD strain, post-use blood cannabinoid levels were significantly higher than pre-use levels for THC (*t*(33) = -4.09, *p*<.001); CBD (*t*(33) = -4.51, *p*<.001; and 11-OH-THC (*t*(33) = -5.66, *p*<.001); but significantly lower for THC-COOH (*t*(33) = -4.19, *p*<.001.

**eResults 2.** Accuracy (d’) additional results

In addition to the significant two-way interaction between strain and cannabis condition, the primary ANOVA investigating strain differences in d’ also revealed a significant two-way interaction of order of van session and cannabis condition (*F*(2, 103) = 4.03, *p* = .047, partial $\eta^{2}$= 0.04); cannabis condition had different effects on d’ depending on order, such that the effect of cannabis condition on d’ was significant when the cannabis van session occurred first, with d’ being greater for the non-cannabis condition than the cannabis condition (difference = 0.27, SE = 0.11, *p* = .01). There also existed a significant two-way interaction between cannabis condition and the number of cannabis use days reported at baseline (the covariate), (*F*(1, 103) = 5.68, *p* = .02, partial $\eta^{2}$= 0.05), such that the slope of the continuous covariate differed depending on cannabis condition, with the slope being steeper (and d’ greater) for the non-cannabis condition than the cannabis condition (difference = 0.02, SE = 0.01, *p* = .02) (see Supplementary Figure 5). Furthermore, the main effect of cannabis condition on d’ was significant, (*F*(2, 103) = 7.68, *p* = .007, partial $\eta^{2}$= 0.07), with d’ being greater for the non-cannabis condition than the cannabis condition (difference = 0.11, SE = 0.08, *p* = .16). This finding should be interpreted in the context of the significant two-way interactions, and should be interpreted with caution, as the mean difference in d’ between non-cannabis and cannabis conditions was not significant. The analysis did not find significant main effects for strain, sex, or van order (*p* > .05).


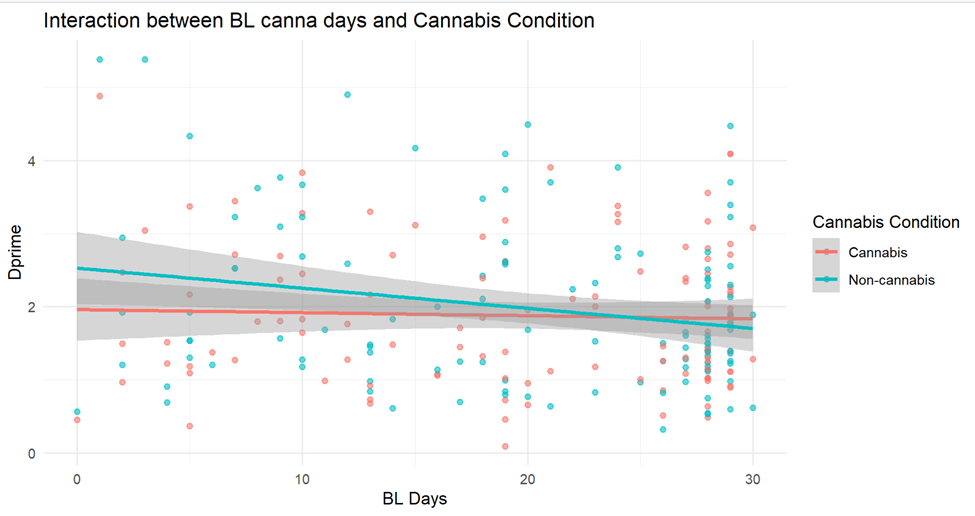


**eFigure 5**. Significant two-way interaction between the number of cannabis use days reported at the baseline appointment (BL Days) and cannabis condition. Chronic effects (i.e., more memory impact for those who used cannabis more prior to baseline) impact acute performance more when participants were in the non-cannabis condition compared to when they were in the cannabis condition. Acute effects may overwhelm chronic effects in the cannabis condition. Tolerance effects may contribute to the flatter cannabis condition slope.

**eResults 3**. Response bias (C) additional results

In addition to the significant two-way interaction between strain and cannabis condition, the main analysis also revealed a significant two-way interaction between order of van session and cannabis condition (*F*(1, 103) = 21.53, *p*<.001, partial $\eta^{2}$ = 0.17). As with d’, the effect of cannabis condition on response bias was significant when the cannabis van session occurred first, with the non-cannabis condition being more conservative than the cannabis condition (difference = 0.21, SE = 0.04, *p*<.001). There was also a significant main effect of strain on response bias (*F*(2, 103) = 3.55, *p* = .03, partial $\eta^{2}$ = 0.06), with the +THC/-CBD strain having a lower average response bias than the +THC/+CBD strain (difference = 0.21, SE = 0.09, *p* = .046). The +THC/-CBD strain did not significantly differ from the -THC/+CBD strain, and the -THC/+CBD strain did not differ significantly from the +THC/+CBD strain. We did not find significant main effects for sex, cannabis condition, or van session order (*p* > .05), and did not observe any other significant interaction terms (*p* > .05).

**eResults 4.** False alarm rate additional results

There was also a significant two-way interaction between order of van session and cannabis condition (*F*(1, 103) = 16.51, *p*<.001, partial $\eta^{2}$= 0.14), with the effect of cannabis condition only being significant when the cannabis van session was first, such that the false alarm rate was greater in the cannabis condition than in the non-cannabis condition (difference = 0.08, SE = 0.02, *p*<.001). The analysis also revealed a significant main effect of strain (*F*(2, 103) = 4.95, *p* = .01, partial $\eta^{2}$= 0.09), such that the false alarm rate was significantly greater for the +THC/-CBD strain than the +THC/+CBD strain (difference = 0.09, SE = 0.03, *p* = .01), though there was no significant difference between the +THC/-CBD strain and the -THC/+CBD strain. Additionally, the -THC/+CBD strain did not significantly differ from the +THC/+CBD strain. There was also a significant main effect of cannabis condition (*F*(1, 103) = 6.53, *p* = .01, partial $\eta^{2}$= 0.06), with the false alarm rate being greater for the cannabis condition than the non-cannabis condition (difference = 0.03, SE = 0.01, *p* = .02). There were no other significant interactions or other significant main effects (*p* > .05).

**eResults 5.** Hit rate additional results

The mixed-methods repeated measures ANOVA revealed a two-way interaction between order of van session and cannabis condition that approached significance (*F*(1, 103) = 3.68, *p* = .058, partial $\eta^{2}$= 0.03). Post-hoc analysis found that when cannabis van sessions occurred first, the hit rate was slightly greater for those in the cannabis condition than in the non-cannabis condition (difference = 0.04, SE = 0.02, *p* = .02), though this should be interpreted with caution as this interaction was not significant at the ANOVA level. There were no other significant interactions and there were no significant main effects (*p* > .05).

**eResults 6.** Reaction time (RT) additional results

The analysis yielded a significant two-way interaction between new words vs. old words and correct responses vs. incorrect responses (*F*(1, 97) = 7.38, *p* = .01, partial $\eta^{2}$= 0.07), such that when responses were correct, reaction time was slower for new words than for old words (difference = 0.04, SE = 0.01, *p*<.001). When responses were incorrect, reaction time was faster for new words than for old words (difference = 0.13, SE = 0.02, *p*<.001). There was a significant main effect of old vs. new words on reaction time (*F*(1, 97) = 4.17, *p* = .044, partial $\eta^{2}$ = 0.04), with a faster reaction time for new words than for old words (difference = 0.04, SE = 0.01, *p*<.001). The main effect of correct vs. incorrect response on reaction time was also significant (*F*(1, 97) = 4.65, *p* = .03, partial $\eta^{2}$= 0.05), with reaction time being slower for incorrect responses than for correct responses (difference = 0.05, SE = 0.01, *p*<.001).

Supplementary Reference

Tucker, D. M. (1993). Spatial sampling of head electrical fields: The geodesic sensor net. *Electroencephalogr. Clin. Neurophysiol.* 87(3):154–163. https://doi.org/10.1016/0013-4694(93)90121-B
